# Unusual Presentation of Extruded Disc: A Case Report

**DOI:** 10.31729/jnma.5993

**Published:** 2021-02-28

**Authors:** Rajesh Pratap Shah, Bishnu Babu Thapa, Sujata Panta, Kabita Birat Karmacharya

**Affiliations:** 1 Department of Orthopaedics, Shree Birendra Hospital, Chhauni, Kathmandu, Nepal; 2 Department of Radiology, Shree Birendra Hospital, Chhauni, Kathmandu, Nepal; 3 Department of Pathology, Shree Birendra Hospital, Chhauni, Kathmandu, Nepal

**Keywords:** *back pain*, *dorsal disc extrusion*, *lumbar herniation*, *unusual presentation*

## Abstract

A 38-year male presented with severe low back pain radiating towards right lower limb which was progressively increasing with decrease in motor power of the ipsilateral ankle dorsiflexion and toe extension. Magnetic Resonance study with gadolinium suggested dorsal epidural migration of the extruded disc at L4-L5 level compressing the thecal sac, which mimics the differential diagnosis epidural abscess, epidural hematoma, synovial cyst and extradural space-occupying lesion. Open lumbar discectomy was done, and the large, herniated disc was found dorsal to the thecal sac adhering dura mater, which was removed meticulously and the patient was symptomatically better postoperatively. The power of his lower limb gradually increased by physiotherapy in subsequent follow-up.

## INTRODUCTION

Lumbar disc herniation is commonly confined to the anterior epidural space. Migration of the disc to the dorsal epidural space is very rare. Only a few cases with sequestrated disc have been reported. We report a very unusual case of migration of extruded disc dorsal to the thecal sac in a 38-year-old male. We review the literature and discuss the difficulty in diagnosis of the extruded disc.

## CASE REPORT

A 38-year-old male presented with a chief complaint of low back pain for six months. The pain was progressive in nature and radiating toward the right lower limb for three months. He was treated conservatively with oral analgesic and physiotherapy, but there was no improvement. The pain gradually increased with the listing of the body towards the left side. On clinical examination, his straight leg raise was 60 degree in both the lower limb, extensor hallucis longus and flexor hallucis longus was 3/5, hypoesthesia in L4 and L5 dermatomes and loss of ankle reflex in the right lower limb. His bowel and bladder habits were normal.

**Figure 1.**
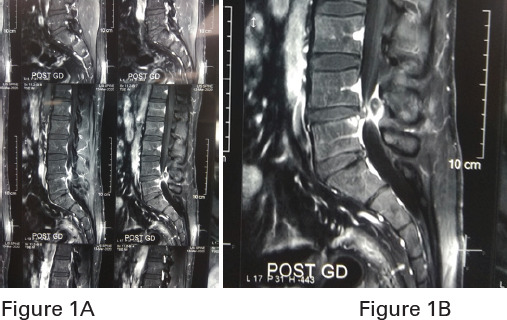
MRI of LS spine showing unusual disc protrusion at L4-L5- 1A: plane, 1B: contrast.

MRI showed a Rt. Posterolateral epidural lesion, measuring approximately 11 x 6.5 x 7 mm in CC x TR x AP axis, was noted at L4-L5 level. The lesion exhibited isointense signal intensity relative to intervertebral disc on both T1 and T2 weighted images and rim enhancement on gadolinium-enhanced T1 weighted image (Figure 1A and 1B). There was a disruption of the outermost annulus of the adjacent disc with disc extrusion and a track like enhancement leading to right posterior epidural space up to the lesion. Laboratory report showed erythrocytes sedimentation rate of 10 and C-reactive protein negative while the rest of other blood parameters were normal.

**Figure 2.**
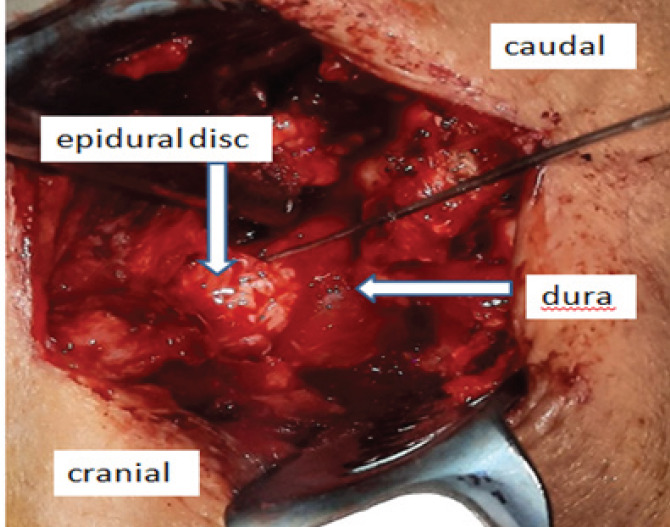
Open lumbar discectomy showing unusual extrusion of disc.

**Figure 3.**
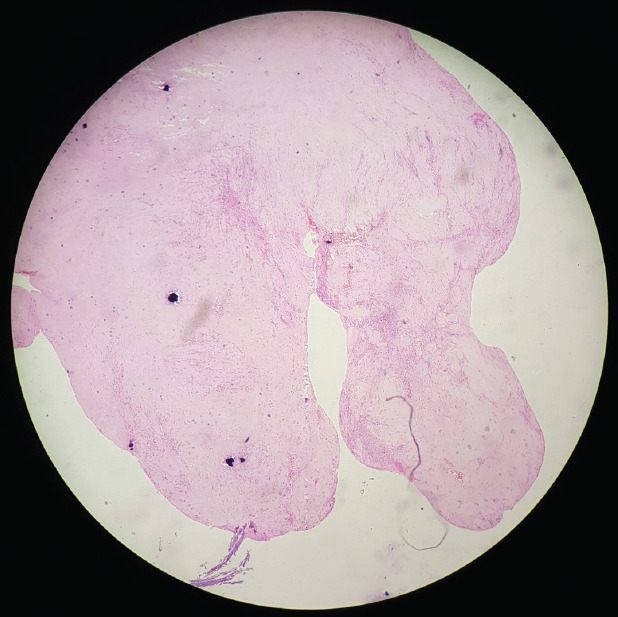
Histopathology of the extruded disc.

Open lumbar discectomy was performed at the L4-L5 level. Large disc material was removed from dorsal epidural space, which was compressing the thecal sac. (Figure 2). Histopathology report of the specimen showed degenerated disc material with myxoid areas, adipocytes and blood vessels with no atypia or evidence of malignancy (Figure 3).

Postoperatively, the patient had relief from back pain and leg symptoms immediately after surgery. The patient was discharged and advised for physiotherapy. His stitches were removed two weeks after the surgery. There was complete recovery of the right lower limb's motor power and sensation during his three months follow up.

## DISCUSSION

Lumbar disc herniation (LDH) is one of the commonest degenerative manifestations of the spine. It is of three types: Protrusion, Extrusion and Sequestration.^1,2^ Disc herniates posterolaterally or posteriorly into the spinal canal. Posterolateral herniation of the disc is the commonest presentation. It is because posteriorly, there is a firm adherent of the posterior longitudinal ligament (PLL) to the periosteum of the vertebral body and the midline septum attaching PLL to the vertebral body hindering the posterior migration. The herniated disc migrates cranially in posterolateral herniation as there is a lack of deep fibres of PLL in the superolateral portion of the intervertebral disc, making more space available to accommodate the herniated disc. In contrast, it migrates caudally in posterior disc herniation. On posterior herniation, it enters into anterior epidural space (AES) by rupturing posterior longitudinal ligaments.^3,4^ However, larger disc fragment herniates into lateral extradural space through the free edge of PLL. It is rear for the herniated disc to reach dorsal epidural space^5-9^ as its migration is limited by a lateral membrane which is attached to PLL medially and wall of spinal canal laterally. Similarly, other structures limiting the dorsal migration of disc in the spinal canal are medial and lateral Dural ligament of Hoffmann, epidural vessel, spinal nerve root, Dura and fat.^5,10^ If there is a failure of any of these structures, the sequestrated disc may migrate dorsally to the thecal sac. There are a few case report stating dorsal epidural migration of sequestrated disc.^5,7-10^ In our case, the extruded disc has reached dorsal epidural space, which is extremely rare. Only one case of dorsal epidural migration of extruded disc has been reported till now in our knowledge, the other being migration of sequestrated disc.^10^ This could be due to sub-ligamentous migration of extruded disc anterior to PLL following the path of least resistance laterally to reach the dorsal epidural space as found preoperatively in our case.

MRI is the investigation of choice for herniated disc and gadolinium enhancement for extruded or sequestrated disc showing peripheral rim enhancement due to neovascularization around the sequestrated disc.^11,12^ The differential diagnosis could be a synovial cyst which is related to facet joint, epidural abscess shows the mass effect with hypointense on T1 and hyperintense on T2, tumor doesn't have rim enhancement.^10,13^ This patient had no constitutional symptoms, and his blood parameters were within the normal limit.

In our case, the disc was extruded at the level of L4-L5 and migrated all the way to dorsal epidural space as suggested in MRI. Gadolinium scan suggested rim enhancement due to neovascularization around the extruded disc suggested by the histopathology report (Figure 3). This type of rare dorsal epidural herniation of the extruded disc could be in case of heavy manual labor, traction therapy for the prolapsed disc, spinal manipulation.^7^ In our case patient was military personnel who had to undergo heave physical exercise.

However, migration of the herniated disc dorsally is one of the extremely rare conditions with the challenge in diagnosis. There are increasing unusual disc herniation cases report, which should make surgeon skeptical while diagnosing extradural spine pathology. However, early surgery is always the treatment of choice to intercept further deterioration of neurological symptoms.
